# Understanding the impacts of drought on peanuts *(Arachis hypogaea* L.): exploring physio-genetic mechanisms to develop drought-resilient peanut cultivars

**DOI:** 10.3389/fgene.2024.1492434

**Published:** 2025-01-08

**Authors:** Sameer Pokhrel, Prasanna Kharel, Swikriti Pandey, Stephanie Botton, Gema Takbir Nugraha, Corley Holbrook, Peggy Ozias-Akins

**Affiliations:** ^1^ Department of Horticulture and Institute of Plant Breeding, Genetics and Genomics, University of Georgia, Tifton, GA, United States; ^2^ United States Department of Agriculture – Agricultural Research Service, Tifton, GA, United States

**Keywords:** drought, peanut, physiology, genetics, breeding, tolerance

## Abstract

Peanut is a vital source of protein, particularly in the tropical regions of Asian and African countries. About three-quarters of peanut production occurs worldwide in arid and semi-arid regions, making drought an important concern in peanut production. In the US about two-thirds of peanuts are grown in non-irrigated lands, where drought accounts for 50 million USD loss each year. The looming threat of climate change exacerbates this situation by increasing erratic rainfall. Drought not only reduces yield but also degrades product quality. Peanuts under drought stress exhibit higher levels of pre-harvest aflatoxin contamination, a toxic fungal metabolite detrimental to both humans and animals. One way to sustain peanut production in drought-prone regions and address pre-harvest aflatoxin contamination is by developing drought-tolerant peanut cultivars, a process that can be accelerated by understanding the underlying physiological and genetic mechanisms for tolerance to drought stress. Different physiological attributes and genetic regions have been identified in drought-tolerant cultivars that help them cope with drought stress. The advent of precise genetic studies, artificial intelligence, high-throughput phenotyping, bioinformatics, and data science have significantly improved drought studies in peanuts. Yet, breeding peanuts for drought tolerance is often a challenge as it is a complex trait significantly affected by environmental conditions. Besides technological advancements, the success of drought-tolerant cultivar development also relies on the identification of suitable germplasm and the conservation of peanut genetic variation.

## 1 Introduction

Peanut (*Arachis hypogaea* L.), also known as groundnut, is an important legume and oilseed crop. It is rich in macronutrients with 40%–50% fat, 12%–36% protein, 10%–20% carbohydrate, and micronutrients including calcium, phosphorus, vitamin E, iron, magnesium, and potassium (Rathnakumar et al., 2017). Peanut is a vital protein source in arid and tropical regions of Asian and African countries, where dietary protein predominantly comes from plant sources such as legumes (Singh and Singh, 1992). The top five peanut-producing countries in the world are China, India, Nigeria, the United States, and Senegal with 38%, 13%, 9%, 6%, and 3%, respectively of global peanut production (USDA-FAS, 2024).

About 70 percent of the total peanut production areas include arid and semi-arid regions where drought has a substantial impact (Reddy et al., 2003). Access to water to mitigate drought is limited in most of the peanut-growing parts of the world. As a result, water and heat stress are major limiting factors to peanut growth, pod yield, and seed quality (Aboelill et al., 2012; Hamidou et al., 2012). In fact, drought is the greatest abiotic stress that reduces the yield of peanuts (Boyer, 1982; Araus et al., 2002). With advancing climate change resulting in erratic rainfall and anomalies in global temperature, it is important to understand how plants can adapt to tolerate these changes. Drought, extreme temperatures, salinity, flooding, and altered CO_2_ concentration are some of the plant abiotic stresses exacerbated in the era of climate change (Onyekachi et al., 2019; Chaudhry and Sidhu, 2022). These environmental aberrations due to climate change influence plant growth and development, their phenology and distribution, and host-pathogen interaction (Coakley et al., 1999; Bertin, 2008; Ghini et al., 2008; Kelly and Goulden, 2008; Raza and Bebber, 2022).

Drought affects plant morphology, physiology, and biochemistry, consequently decreasing plant production (Seleiman et al., 2021). Developing drought-tolerant peanut cultivars can offer a sustainable solution for utilizing scarce water to produce higher yields in drought-prone peanut production areas. It is crucial to dissect the physiological and genetic mechanisms underlying drought tolerance to develop a successful drought-tolerant peanut variety. This review paper discusses the negative effect of drought on peanut production and seed quality and delves into the physiological parameters that enable drought-tolerant peanut cultivars to perform superior to susceptible cultivars in water-stressed conditions. It further explores the genetic basis of drought tolerance, examining the molecular mechanisms by which drought is detected and signaled, and how the interaction of genes, hormones, and several other biochemicals contribute to drought tolerance. The current progress in the utilization of genetic engineering, exploration of the peanut germplasm from different genetic pools, molecular breeding, and technological advancements that successfully developed drought-tolerant cultivars have also been elucidated. Based on the recent achievements, insights have also been drawn on the potential research areas for future peanut genetic improvements.

## 2 Effects of drought on peanut yield and quality

Drought has a detrimental effect on peanut production resulting in as much as 85% reduction in pod yield (Nageswara Rao et al., 1988). It severely diminishes the uptake of nutrients such as nitrogen, phosphorus, and potassium. Consequently, peanut growth and development are affected, resulting in reduced biomass, pod yield, and quality (Reddy et al., 2003; Junjittakarn et al., 2013; Htoon et al., 2014; Nageswara Rao et al., 1985; Aydinsakir et al., 2016). Along with reduction in pod yield, the seeds produced by water-stressed peanut plants also have poor grades, vigor, and viability (Pallas Jr et al., 1979).

The severity of drought on peanut plants is dependent on the timing, duration, and intensity of drought stress (Hamidou et al., 2012). At different developmental stages, like flowering, peg initiation, and seed maturity, peanut yield was found to vary under drought stress (Nageswara Rao et al., 1988; Hamidou et al., 2013). Reduced irrigation causes the soil to dry up making peg penetration for pod formation difficult. Even when the pegs penetrate the soil, reduced moisture in the root zone may hinder pod formation. In contrast, reduced water supply at the pre-flowering stage followed by adequate watering causes the plants to initiate a flush of flowering which results in increased pod yields by 13%–19% as compared to peanuts with regular irrigation regimes (Nageswara Rao et al., 1985; Nautiyal et al., 1999; Puangbut et al., 2010). However, prolonged drought from flowering initiation to seed filling stage results in up to 50% yield reduction (Stansell and Pallas, 1985). This information could be important in planning water regimes to increase irrigation efficiency and reduce drought stress.

Besides yield, drought stress also negatively impacts the nutritional quality of peanut seeds. The impact is particularly observed in the composition of fatty acids. High oleic acid composition is advantageous not only for enhancing flavor and nutritional value but also for extending shelf-life. The ratio of oleic to linoleic (O/L) in conventional US varieties is 1.5–2.4 (Dwivedi et al., 1996; Davis and Dean, 2016). The impact of drought on the O/L ratio has been conflicting over the years. Some studies report increased O/L ratio at the end-of-season drought in peanut seeds (Dwivedi et al., 1993; Dwivedi et al., 1996; Chaiyadee et al., 2013) while others report decreased O/L ratio (Hashim et al., 1993). The overall oil content can decrease when peanuts encounter mid-season or end-of-season drought (Conkerton et al., 1989; Dwivedi et al., 1996). While the oil content decreases at the end of the season drought, it can result in the increased protein content at the expense of oil content (Dwivedi et al., 1996).

Prolonged periods of drought and heat stress can cause increased aflatoxin contamination in peanut seeds deteriorating their quality (Hamidou et al., 2014). Aflatoxin B1 produced by *Aspergillus flavus* is highly carcinogenic to both animals and humans, resulting in suppressed immunity, malnutrition, and cancer. As a result, peanuts produced for consumption and commercialization are closely monitored all over the world (Cole et al., 1995; Girdthai et al., 2010; Yu, 2012; Hou et al., 2014; Holbrook et al., 2016). USDA permits no more than 15 ppb (parts per billion) of aflatoxin in any peanut products while the European Union limits it to 4 ppb (Office of the Federal Register, 2004; European Commission, 2024). Elevated temperatures and lowered soil moisture have been linked to an increase in *Aspergillus* populations in the soil. If the environmental conditions persist, the fungus can infect pods and seeds leaving growers and producers with unusable and potentially aflatoxin-contaminated peanuts. This is more pronounced if there is a moisture deficit during the pod-filling stage (Arunyanark et al., 2009).

Development and utilization of drought tolerant peanut cultivars can help ameliorate aflatoxin contamination. Girdthai et al. (2010) observed that drought-tolerant cultivars had lower *Aspergillus* colonization and pre-harvest aflatoxin contamination. Holbrook et al. (2000) also reported a similar trend where some genotypes that avoided drought exhibited 92% lower aflatoxin contamination as compared to a check cultivar. Since aflatoxin contamination in peanut genotypes is highly dependent on the genotype by environment interaction, the studies that correlate drought stress and aflatoxin contamination are not always consistent (Hamidou et al., 2014). Drought stress and aflatoxin contamination are closely related and there is a need to dissect genetic mechanisms for drought stress to understand its relation to aflatoxin contamination.

## 3 Morpho-physiological mechanisms of drought tolerance

**FIGURE 1:**
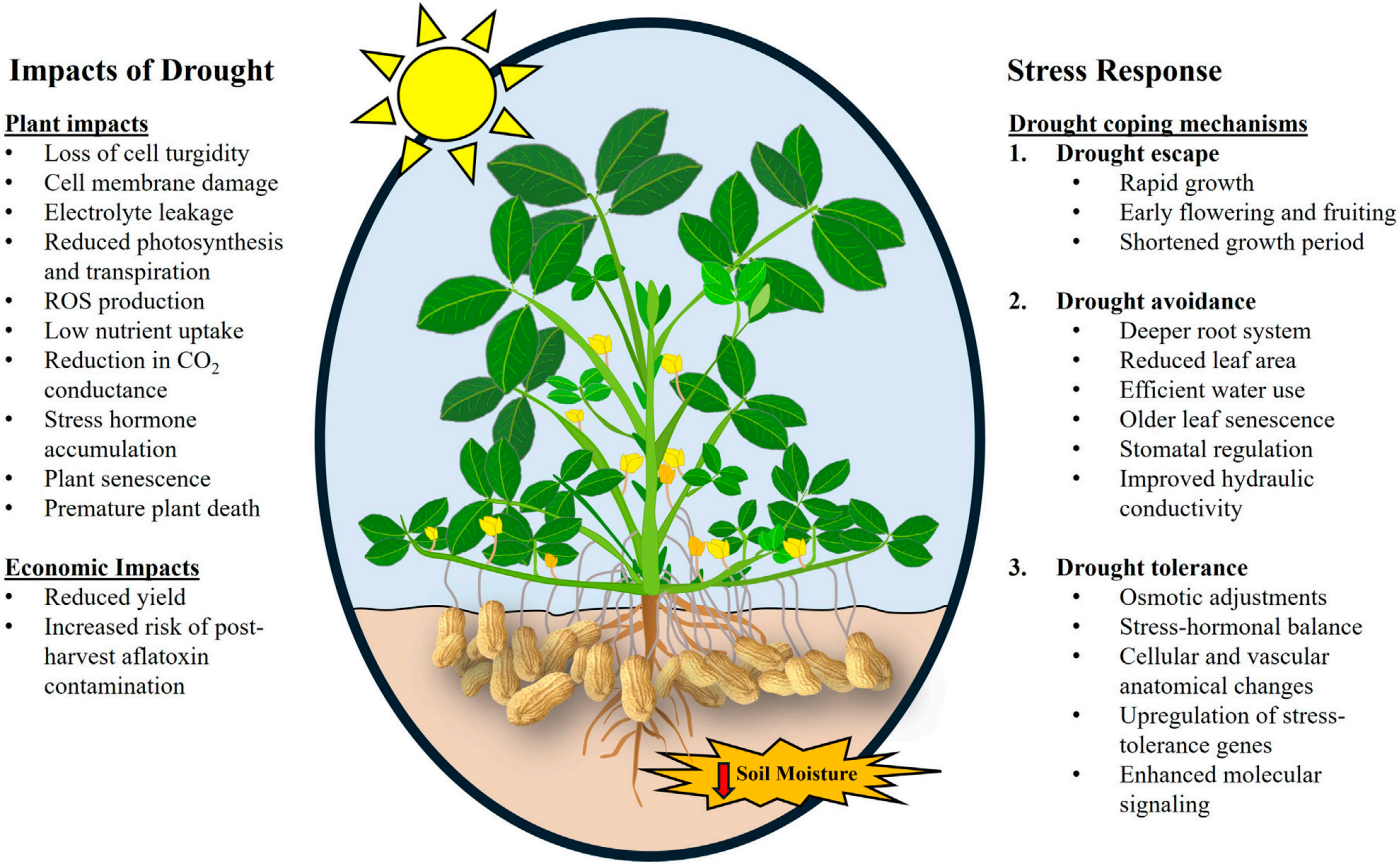
Image showing the impacts of drought in peanuts and tolerance mechanisms of drought-tolerant peanut cultivars. Image modified from https://www.ruralsprout.com/grow-peanuts/.

Drought elicits various physiological and Morphological changes in peanuts that constrain its growth and development. The changes are often exhibited as a decrease in water content, cell expansion and division, nutrient absorption, and compromised photosynthetic capability (Hou et al., 2014). In response, peanuts exhibit escape, avoidance, and tolerance strategies to cope with drought stress (Figure 1). Escape refers to strategies such as early flowering, early maturing, and short growth periods where plants complete the life cycle before the onset of stress. Avoidance strategies include structural or physiological adaptations that reduce plants exposure to drought stress, such as thicker waxes or numerous hairs, larger or deeper root systems, efficient use of water for survival, and limited productivity. Tolerance is the ability of plants to endure stress conditions through survival mechanisms such as plant protection gene induction, vascular and anatomical changes, and osmotic adjustment (Bueckert and Clarke, 2013; Dang et al., 2013).

Root architecture and physiology can play a significant role in coping with drought stress. Peanuts grown under water-deficit conditions have longer and thicker roots, which increases the surface area and enhances their capacity to access moisture in water-stressed conditions (Rowland et al., 2012). Peanuts exhibited a greater root density at 0.4–0.8 m soil depth during water deficit as a strategy to cope with drought (Pandey et al., 1984). Songsri et al. (2008) also observed similar results, where drought-tolerant peanut cultivars had increased root length density (total root length/soil volume) at 40–100 cm deep soil, which helped them avoid drought stress and maintain pod yield. Anatomical dissections of peanut roots under drought conditions showed a decrease in the area of xylem vessels (Thangthong et al., 2018). This trait in drought-tolerant cultivars helps the plant to improve water uptake and reduces the risk of xylem cavitation (bubble formation). As a leguminous crop, peanut roots develop root nodules which help in fixing atmospheric nitrogen into a usable form for plants. Several studies support the fact that roots of drought-tolerant peanut cultivars have a higher nitrogen fixation ability than the susceptible cultivars in a drought-stressed environment (Pimratch et al., 2008; Dinh et al., 2013; Furlan et al., 2017).

Besides roots, leaf morphological and anatomical modifications can also be observed in peanuts tolerant to drought stress. Sankar et al. (2013) reported reduced leaf thickness, epidermis, and number of cells in the palisade and spongy regions when peanuts were subjected to drought stress. Peanut cultivars with greater drought tolerance had greater leaf thickness, higher palisade to spongy tissue ratio, specific leaf weight (dry weight per unit leaf area), and leaf area compared to drought susceptible cultivars (Li et al., 2014).

Drought tolerance in peanuts is also determined by stomatal regulation, where stomatal opening/closing is adjusted to minimize water loss. Stomata are microscopic pores on leaves and stems that allow and regulate the gaseous exchange, influencing photosynthesis and controlling water loss during transpiration (Richardson et al., 2017; Driesen et al., 2020). As plants adjust stomatal aperture to minimize water loss during water stress, such stomatal closure reduces the gaseous exchange and photosynthesis rate, affects plant growth and development, and builds up harmful oxidative molecules (Chaves et al., 2008; Evans, 2013; Gururani et al., 2015; Pirasteh-Anosheh et al., 2016). Soba et al. (2024) observed that the earliest reactions to water stress were reduction in stomatal conductance and photosynthesis. As the drought progressed, a reduction in fluorescence parameters and later changes in biochemical traits impacting carbon fixation were observed. Although stomatal conductance is a common indicator of drought stress in peanuts, the decline in the net photosynthesis is mostly attributed to other metabolic constraints such as disruption of thylakoid processes and limitation in electron transport through photosystem II (Pilon et al., 2018). Drought-tolerant peanut cultivars can cope with water scarcity by closing the stomata faster during water stress (Jiang et al., 2021) and can also maintain photosynthesis efficiency enhancing yield in limited water conditions (Zhen et al., 2022).

Plants accumulate osmolytes which help them in the osmotic adjustments, enable water uptake, maintain cellular homeostasis, and facilitate redox mechanisms by removing excess reactive oxygen species (Ghosh et al., 2021). During drought stress, drought-tolerant peanut cultivars accumulate several osmolytes to significantly higher levels than the drought susceptible cultivars (Celikkol Akcay et al., 2010; Padmavathi and Rao, 2013). Such osmolytes include proline, soluble sugar, free amino acids, and soluble proteins (Zhang et al., 2017; Furlan et al., 2020). Gundaraniya et al. (2020) mentioned that 46 different drought-responsive metabolites including polyamines such as agmatine and cadaverine were present in the roots and leaves of drought-tolerant peanuts. Drought-tolerant peanuts maintain the integrity and stability of cell membranes under drought stress by utilizing antioxidative defenses such as catalase and ascorbate peroxidase along with proline to prevent cellular oxidative damage (Celikkol Akcay et al., 2010). This is important as the cell membrane is one of the primary targets of plant stressors and water stress can damage the cell membrane resulting in the leakage of cellular electrolytes limiting the photosynthesis (Agarie et al., 1995; Lauriano et al., 2000; Bajji et al., 2002).

Physiological traits such as specific leaf area (SLA, ratio of leaf area to dry weight), specific leaf nitrogen (SLN, the ratio of nitrogen content to leaf area), harvest index (HI, the proportion of economic yield in total biomass), water use efficiency (WUE, amount of carbon assimilated as grain/biomass per unit water used), soil plant analysis development (SPAD) chlorophyll meter reading (SCMR, indication of light-transmittance characteristics of the leaf dependent on the leaf chlorophyll content) are indicators of drought resistance/susceptibility in peanuts. Traits such as WUE are difficult to measure on a large scale hence surrogate traits, such as SLA and SCMR, are often used in peanut research. It has been established that SCMR and WUE are correlated, indicating the possibility of using SCMR as a surrogate trait to screen WUE in large populations (Nageswara Rao et al., 2001; Nigam et al., 2005). SCMR was also observed to be strongly correlated to pod yield (Upadhyaya, 2005). Traits such as leaf area index (LAI), SCMR, transpiration efficiency, relative water content (RWC), and canopy temperature (CT) have been used as surrogate traits to study drought tolerance in peanuts (Krishnamurthy et al., 2007; Shaibu et al., 2020). LA is also associated with RWC. Drought-stressed peanut plants have lower RWC (30%) than non-stressed plants (85%–90%) (Reddy et al., 2003; Kambiranda et al., 2011). Nautiyal et al. (2002) observed that drought tolerance was higher in peanut cultivars with low SLA as they maintained higher RWC, suggesting low SLA peanut cultivars are more water-use efficient. Transpiration efficiency (TE) measured using lysimeters in peanuts found that high TE increased pod yield during intermittent drought under dry and hot conditions but was less pronounced under more wet and cool conditions (Vadez and Ratnakumar, 2016). The study demonstrated that TE was not correlated with its commonly used surrogate traits (SCMR and SLA) and hence a direct TE rather than its surrogate evaluation was suggested. The impacts of drought on peanuts have also been illustrated in Figure 1.

## 4 Molecular and biochemical dissection of drought tolerance

The molecular and biochemical basis of drought stress perception and response has not been fully elucidated in peanuts (Bhogireddy et al., 2020; Jiang et al., 2021). Therefore, insights from studies on model plants and other crops need to be applied to understand the mechanism of drought tolerance in peanuts. Broadly, drought tolerance occurs through two main steps: drought stress signal reception and transduction followed by initiation of stress response (Ul-Allah et al., 2021). These responses can occur at physiological, morphological, biochemical, or molecular levels (Mahmood et al., 2019). The physiological and morphological basis for tolerance has been discussed in the previous sections. In this section, the molecular and biochemical basis of stress tolerance in plants is discussed followed by the studies specific to drought tolerance in peanuts. It concludes with the review of biotechnological approaches applied to develop drought-tolerant peanuts.

### 4.1 Drought reception and response

It is imperative to understand the fundamental mechanisms by which plants sense and respond to drought stress, from initial stress perception to final adaptive responses. This knowledge is crucial for guiding informed efforts to breed drought-tolerant crops, including peanuts (Huang et al., 2012; Shaibu et al., 2020). Significant effort has been invested to elucidate the complex mechanism of drought signaling and response in many plants, yet there are a lot of unknowns to decipher (Takahashi et al., 2020; Yang et al., 2021). Some of the mechanisms for drought sensing and response shown by the plants may be common with other abiotic stresses such as cold, and salt stress (Shinozaki and Yamaguchi-Shinozaki, 2000; Huang et al., 2012). In general, upon stress perception by the cell membrane receptors, many intracellular secondary messengers like Ca^+2^, and reactive oxygen species (ROS) are released (Shinozaki and Yamaguchi-Shinozaki, 2000; Huang et al., 2012). These secondary messengers, with the help of different protein kinases and protein phosphatases, activate the transcription factors which then induce the expression of certain genes in response to stress (Huang et al., 2012).

The initial signal of water deficiency originates in the roots when the roots perceive decreased water potential in the soil as a dehydration stress (Christmann et al., 2013; Takahashi et al., 2018a). This signal is then transmitted to the leaf tissue via several intercellular messenger signals, including hydraulic pressure, ROS/Ca^+2^ waves, and peptide signals such as Clavata3/Embryo-Surrounding Region-Related 25 (CLE25) peptide (Takahashi et al., 2018b; Takahashi et al., 2020). Upon recognition of these signals by the receptors in the cell membranes of the leaf cells, intracellular secondary messengers such as ROS and Ca^+2^ amplify and transmit the signal. This leads to several biochemical, physiological, and molecular changes in the tissue (Lata et al., 2011; Nataraja and Parvathi, 2016; Yang et al., 2021). The most important change is the increase in the level of abscisic acid (ABA) in the cells (Lata et al., 2011; Takahashi et al., 2020; Yang et al., 2021).

**FIGURE 2:**
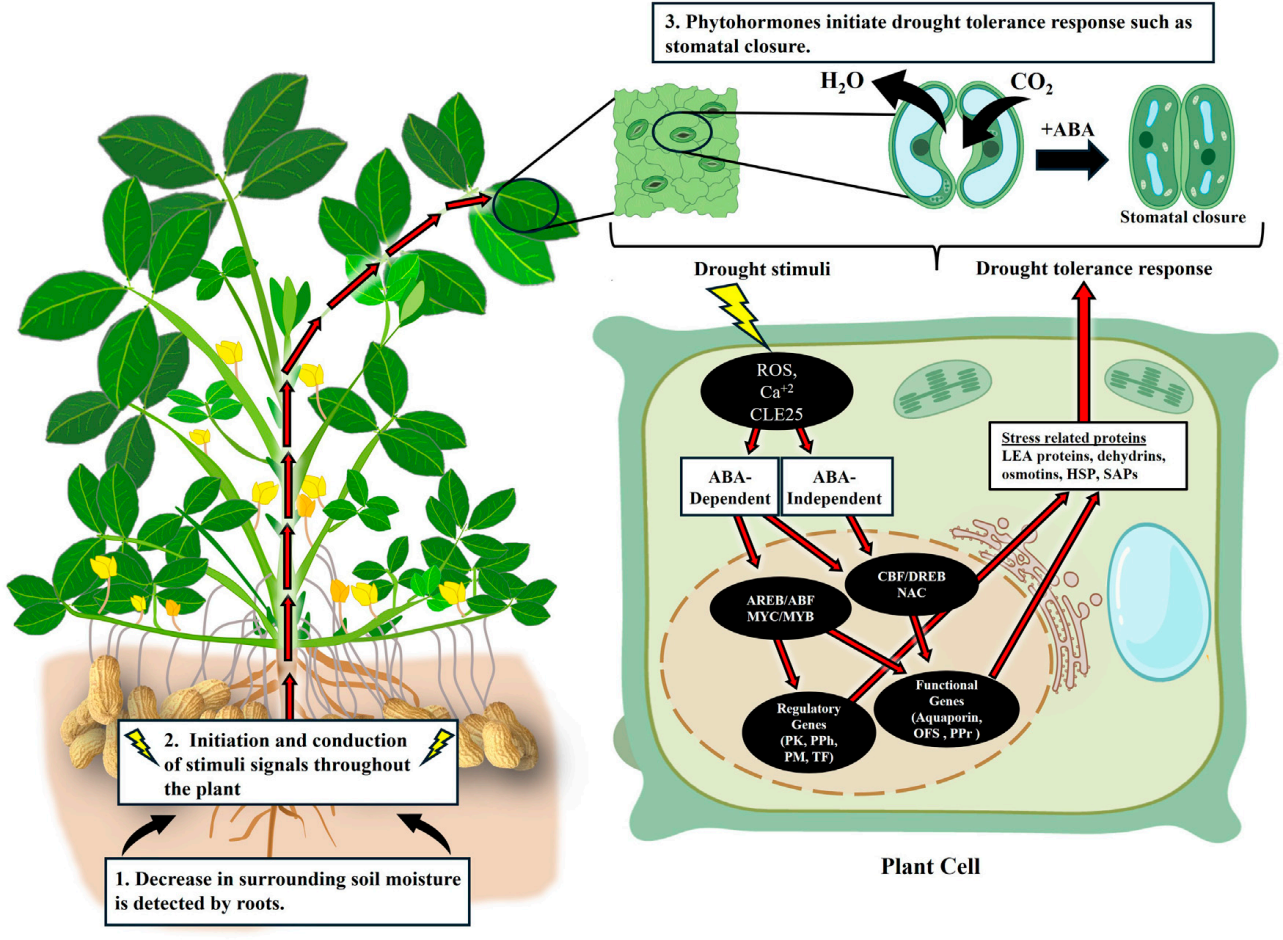
Image showing the molecular mechanism of drought tolerance, from drought perception to activation of response related to drought tolerance. Image modified from Biorender.com and stress related protein listed from Priya et al. (2019). ROS, Reactive Oxygen Species, CLE25, Clavata3/Embryo Surrounding Region-Related 25, AREB/ABF, ABA-Responsive Element-Binding Protein/ABA Binding Factor, MYC/MYB, Myelocytomatosis Oncogene/ Myeloblastosis Oncogene, PK, Protein Kinase genes, PPh, Protein Phosphate genes, PM, Phospholipid Metabolism-related genes, TF, Transcription Factors, OFS, Osmoregulatory Factor Synthase genes, PPr, Protective Protein genes, LEA, Late Embryogenesis-related proteins, HSP, Heat Shock Proteins, SAPs, Stress-Associated Proteins.

Accumulation of ABA is sensed by the stomatal guard cells which induce stomatal closure preventing further loss of water through transpiration (Figure 2). While ABA is the primary hormone regulating stomatal closure, other hormones, including auxin, cytokinin, ethylene, brassinosteroids (BRs), jasmonic acid (JA), and salicylic acid (SA), can act synergistically or antagonistically in this process (Khan et al., 2020; Bandurska, 2022). In addition to their role in stomatal closure, they also affect the morphology and growth patterns of shoots and roots (Chen et al., 2006; Pierik et al., 2007; Zhou et al., 2018; Zhang et al., 2022b). During drought, auxin and cytokinin levels decline in leaves and shoots but accumulate in roots (Havlová et al., 2008; Xu et al., 2016; Abuzeineh and Abualia, 2023). This causes reduction in the shoot growth and an increase in primary root growth by suppressing the lateral root growth. The role ethylene plays in drought response may vary and can be species dependent (Wilkinson and Davies, 2010; Müller, 2021). JA has been shown to act synergistically with ABA to provide drought response while the role of SA and BRs in drought response is still arguable (Zhao et al., 2023; Yoshida and Fernie, 2024). The complex crosstalk between these hormones complicates understanding the role of individual hormones in drought tolerance (Wilkinson et al., 2012).

Although stomatal closure prevents water loss, it disrupts photosynthesis. This intensifies ROS production in the cells due to photorespiration, electron leakage in the Mehler reaction, and reduction in the Calvin cycle (Helena and Carvalho, 2008). ABA also contributes to ROS accumulation and stimulates the plant’s antioxidant defense system in a positive feedback loop (Jiang and Zhang, 2002; Mittler and Blumwald, 2015). Under normal conditions, the oxidative effect of these ROS is balanced by the enzymatic and non-enzymatic antioxidant defense systems of the plants (Sachdev et al., 2021). The enzymatic defense system includes genes like superoxide dismutase (SOD), catalase (CAT), and ascorbate peroxidase (APX), whereas the non-enzymatic system includes vitamins, carotenoids, and flavonoids (Hasanuzzaman et al., 2020). However, the increased production of ROS caused by drought stress can overwhelm the plants antioxidant capacity leading to oxidative damage of the cells (Miller et al., 2010). To mitigate these effects, expression of multiple drought-responsive genes is crucial.

Upon drought stress signal perception and transduction, plants activate their response to drought through ABA-dependent and ABA-independent pathways (Shinozaki and Yamaguchi-Shinozaki, 2000; Takahashi et al., 2018a) (Figure 2). In the ABA-dependent pathway, ABA directly participates in the expression of the drought stress responsive genes (Soma et al., 2021). The increased level of ABA inside the cells due to drought stress leads to binding of ABA with the ABA receptor, pyrabactin resistance1/pyr1-like/regulatory components of ABA receptor (PYR/PYL/RCAR), forming an ABA-PYR/PYL/RCAR complex. This complex then interacts with protein phosphatase 2C (PP2C). Under normal conditions, PP2C prevents the activation of subclass III sucrose non-fermenting 1-related protein kinases 2 (SnRK2s). Due to the interaction of the ABA-PYR/PYL/RCAR complex and PP2C, SNRK2s are released and become activated either by autophosphorylation or phosphorylation in the presence of other kinases. These activated SNRK2s can phosphorylate transcription factors such as ABA-responsive element-binding protein/ABA binding factor (AREB/ABF) and the myelocytomatosis oncogene/myeloblastosis oncogene (MYC/MYB), which then regulate the expression of drought stress responsive genes (Lata et al., 2011; Soma et al., 2021).

The ABA-independent pathway is activated during the early stages of drought stress before sufficient ABA accumulation occurs to trigger the ABA-dependent pathway (Soma et al., 2021). The osmoreceptors that perceive the drought stress and induce downstream responses are not clear (Soma et al., 2021); however, calcium, JA, and ROS, have been linked to ABA-independent drought sensing and responses in cotton (Mahmood, 2020). This pathway also contributes to the early production of ABA by upregulating the expression of the *9-cis-epoxycarotenoid dioxygenase 3* (NCED3) gene. Unlike the ABA-dependent pathway, which relies on subclass III SnRK2 protein kinases, the ABA-independent pathway employs subclass I SnRK2 protein kinases.

In the initial stages of osmotic stress, B4 Raf-like kinases phosphorylate the subclass I SnRK2s and activate them. In addition, the activity of subclass I SnRK2s is also influenced by stress-induced accumulation of phosphatidic acid (PA), which is regulated by phospholipase Dα (PLDα1) gene. The activated subclass I SnRK2s phosphorylate the mRNA decapping activator VARICOSE (VCS), which degrades mRNA by removing the 5′ cap. This interaction of subclass I SnRK2s and VCS forms the SnRK2-VCS signaling module which positively regulate the expression of stress responsive genes by controlling the post-translational mRNA population (Soma et al., 2021). Even though the ABA-independent pathway is not fully understood, transcription factors like cold-binding factor/dehydration-responsive element binding (CBF/DREB), NAC (NAM, ATAF, and CUC) and ZF-HD (zinc-finger homeodomain) have been associated in the regulation of drought responsive genes in the absence of ABA (Saibo et al., 2009; Lata et al., 2011).

These two pathways do not work mutually exclusive of each other. There exists a crosstalk between ABA-dependent pathways and ABA-independent pathways to coordinate drought stress responses (Figure 2) (Nakashima et al., 2014). For instance, some NAC transcription factors and DREBs have been reported to participate in both ABA-dependent and independent pathways (Lata and Prasad, 2011; Liu S. et al., 2018). This highlights the complex network underlying drought stress signal transduction and expression of drought responsive genes.

The activation of the transcription factors triggers the activation and expression of the stress-responsive genes. The drought stress-responsive genes can be categorized into two categories: functional genes, the products of which are directly engaged in resisting drought stress and providing stress tolerance, and regulatory genes, which indirectly respond to drought stress via signal transduction and regulation of gene expression (Huang et al., 2012; Yang et al., 2021). Functional genes include those encoding aquaporins, osmoregulatory factor synthases, and protective proteins. In contrast, regulatory genes encompass protein kinase genes, protein phosphatase genes, phospholipid metabolism-related genes, and transcription factor genes (Shekoofa and Sinclair, 2018; Mahmood, 2020; Yang et al., 2021).

### 4.2 Identification of genes associated with drought tolerance in peanut

As mentioned earlier, comprehensive studies that describe the entire process from drought perception by the peanut plant leading up to the responses against the drought stress are still limited. This section details studies conducted on peanuts to identify genes related to drought. Earlier studies on genes associated with drought tolerance in peanuts focused on a few genes that were already discovered in model crops. Later, with advancements in sequencing technologies, several transcriptomic and metabolomic studies have been conducted on peanuts.

One of the earliest studies to identify genes contributing to drought tolerance in peanuts was the characterization of the phospholipase D (PLD) gene (Guo et al., 2006). PLD gene encodes phospholipase D enzyme associated with lipid degradation and drought stress signal transduction (Katagiri et al., 2001; Wang et al., 2014). Guo et al. (2006) associated the role of PLD enzyme in drought sensitivity and tolerance in peanuts which was further confirmed by other studies (Dramé et al., 2007; Zhang et al., 2023). An early increase in PLD accumulation was detected in susceptible peanut cultivars leading to membrane degradation, while a higher accumulation of protective proteins like “late embryogenesis abundant” (LEA) was observed in drought-tolerant cultivars (Guo et al., 2006; Dramé et al., 2007). Two highly expressed genes, serine-rich protein (AhSrp) and leucine-rich protein (AhLrp), were discovered during water stress in drought-tolerant peanut cultivars (Devaiah et al., 2007). These genes encode proteins containing a signal peptide which likely show their participation in drought stress signaling.

An extensive study on the preferential expression of drought-induced genes under gradual water stress in peanuts established the involvement of several upstream signaling components, such as calmodulins, G protein, and receptor kinases, during drought stress (Govind et al., 2009). These signaling components are the core regulatory genes linked with drought signal transduction in peanuts. They also reported induction of several regulatory transcription factors such as NAC, basic helix-loop-helix (bHLH), APETALA 2/ethylene-responsive element binding factor (AP2/ERF), basic leucine zipper (bZIP), CCAAT box, Homeobox, Jumonji, and several zinc finger protein genes under water stress. These transcription factors are components of the ABA-dependent and ABA-independent pathways as mentioned in the earlier section. The presence of ABA-dependent and ABA-independent pathways for drought tolerance in peanuts was further confirmed through a transcriptome analysis on peanut seedlings under water deficit conditions in the presence or absence of ABA pretreatment (Li et al., 2014). Subsequent research showed upregulation in the expression of genes (like AhNCED1, AhZEP, AhBG12, AhBG24, AhAAO2, AhABA3) linked to ABA production, and genes (such as AhABCG22.1 and AhABCG22.2) linked to ABA transport under drought conditions (Long et al., 2019). These studies provide evidence for the presence of ABA-dependent and ABA-independent pathways for drought tolerance in peanuts like other plants as mentioned earlier.

Several studies in peanuts have confirmed the role of transcription factors in regulation of drought responsive genes. The role of AP2/ERF in drought response in peanuts was confirmed through the study involving drought stress and drought release conditions (Dang et al., 2012). Yuan et al. (2020) studied the NAC transcription factor gene family in cultivated and diploid wild peanuts and characterized 164 NAC proteins in cultivated peanuts. During drought stress, 39 of those genes were upregulated, and 13 genes were downregulated. Zhao et al. (2020) evaluated the WRKY gene family in peanuts under drought treatments and reported 158 AhWRKY genes out of which 73 were differentially expressed. These studies demonstrate the involvement of genes and their interactions contributing to the complexity of molecular dissection of drought tolerance in peanuts.

In addition to the transcription factors, genes encoding functional proteins contribute to drought tolerance in peanuts. Genes for heat shock proteins (HSPs), DnaJ-like proteins, aldehyde reductase, proline amino peptidase, choline kinase, defensins, and so on, have been described to play a role in drought tolerance in peanuts (Govind et al., 2009; Pruthvi et al., 2013). Enhanced expression of proline biosynthesis genes (P5CS1, P5CS2a, P5CS2b, P5CR) and relatively lower expression of proline catabolism genes (ProDH1, ProDH2) has been linked to drought tolerance in peanut cultivars under drought stress conditions (Furlan et al., 2020). Hou et al. (2014) also identified functional genes involved in drought response, including those related to stress and hormone signaling, membrane transport, and photosystems.

Protein expression analysis in peanut leaf tissue under drought stress showed five major classes of proteins: signal transduction proteins (e.g., calcium ion binding protein), molecular chaperones (e.g., LEA-1 and HSP protein), photosynthetic proteins (e.g., photosystem I (PSI) proteins), defense proteins (e.g., lectins), and detoxification proteins (e.g., APX-1) (Thangella et al., 2018). This shows that a wide range of drought responsive proteins are necessary to provide drought tolerance in peanuts. A study on the metabolomic profiles of two peanut genotypes with varying drought tolerance, conducted under simulated drought conditions using polyethylene glycol-6000 (PEG-6000), revealed an increase in sugars, organic acids, alcohols, and fatty acids in response to drought stress (Gundaraniya et al., 2020). These compounds include mannose, pentitol, cinnamic acid, caffeic acid, salicylic acid, and fatty acid desaturase enzymes, which provides evidence that a broad range of organic compounds are associated with drought tolerance mechanisms in peanuts. A transcriptome analysis of two peanut cultivars with contrasting drought tolerance traits under fully irrigated and water deficit conditions showed more than 4,500 differentially expressed genes associated with 104 pathways. These pathways were involved in the biosynthesis of plant antibiotics, amino acids, and flavonoids or metabolism of sugars and amino acids (Bhogireddy et al., 2020). The same study revealed that differences in the levels of ABA and expression of sucrose metabolic pathway genes determined their drought tolerance phenotypes. Clearly a large number of genes involved in multiple pathways work in synergy to provide tolerance to drought in peanuts.

In addition to genes and their products, microRNAs (miRNAs), small non-coding RNAs typically 20–24 nucleotides in length, play a role in enhancing drought tolerance in many crops including peanuts (Ferdous et al., 2015). The miRNAs can lead to translation inhibition/mRNA degradation of the target genes resulting in enhanced stress tolerance (Singh et al., 2023). In a bioinformatic evaluation, 33 conserved and 33 novel candidate miRNA families associated with drought and heat stresses were identified in peanuts (Mittal et al., 2023). Ren et al. (2021) identified 73 miRNAs differentially expressed in drought-tolerant peanut variety NH5 under drought stress, of which, novel miR_73 and novel miR_416 were identified as key miRNAs linked to drought adaptation. Functional analysis of miRNAs and their target genes under drought identified miR2111 and miR482 families that targeted genes like lipid transfer protein (LTP) and NAC transcription factor, involved in drought tolerance in peanuts (Zhang et al., 2017). Root tissues were isolated from ‘C76-16’, a drought-tolerant peanut cultivar, to study the mRNA and miRNA expression, which identified 350 differentially expressed miRNAs under drought and revealed that miRNA target genes were mostly linked to redox reactions, modification of cell wall, and transcription regulation during drought stress and drought adaptation (Kumar, 2017).

Above studies indicate the presence of biochemical and molecular pathways involved in drought tolerance in peanuts. While not comprehensive, they have contributed to the partial understanding of molecular basis of drought tolerance in peanuts. Similar to other plants, Ca+2 ions, ROS, and different kinases are involved in drought signal perception and transduction in peanuts (Bhogireddy et al., 2020). Once the drought signal is perceived, the ABA-dependent and ABA-independent pathways are triggered (Li et al., 2014). This activates numerous transcription factors which enhance the peanut’s response to drought by regulating the expression of several functional and regulatory genes. Further studies are required to unravel the complete sequence of events, from initial drought perception to the development of drought tolerance, to fully understand the molecular mechanism of drought tolerance in peanuts.

### 4.3 Genetic engineering for drought tolerance

Most of the drought-related transgenic studies in peanuts employ *Agrobacterium-*mediated transformation and have utilized genes from many organisms to study their response in peanuts (Krishna et al., 2015; Mallikarjuna et al., 2016). These studies have mostly focused on overexpressing genes. Despite the prospect of CRISPR/Cas9[clustered regularly interspaced short palindromic repeats (CRISPR)/CRISPR-associated protein 9 (Cas9)] for peanut genetic improvement, there are no CRISPR/Cas9 genome editing studies on peanuts published for drought tolerance till date (Puppala et al., 2023).

The first transgenic peanut line for drought tolerance was developed by Bhatnagar-Mathur et al. (2007) when transcription factor DREB1 was introduced from *Arabidopsis thaliana* under the control of stress-inducible rd29A promoter into a drought sensitive peanut cultivar (Sun et al., 2013). These transgenic peanut lines exhibited increased transpiration efficiency under water deficit conditions indicating that they were more drought tolerant compared to the wild types. Such increased transpiration efficiency and better performance was due to enhanced root growth and their ability to extract water from deeper soil depths in water deficit conditions (Vadez et al., 2007; Vadez et al., 2013). Further evaluation of these lines in field conditions for 4 years confirmed that these transgenic lines consistently yielded better under drought stress compared to wild types (Bhatnagar-Mathur et al., 2014). In a different study, DREB1A transgenic peanuts, produced similarly as Bhatnagar-Mathur et al. (2007), were characterized (Sarkar et al., 2014). These lines were reported to exhibit higher osmotic potential, proline content, leaf chlorophyll content, photosynthesis ability, and shoot biomass compared to wild type, which led to enhanced performance under drought stress. Another study on peanut lines overexpressing DREB1A demonstrated higher transpiration efficiency, increased antioxidant enzymes, and higher proline synthesis, which collectively contributed to better drought tolerance in transgenic lines than wild type lines (Bhalani et al., 2019).

Multiple studies have overexpressed either single or multiple regulatory genes to improve the drought tolerance capacity in transgenic peanut lines. Overexpressing NAC transcription factor (MuNAC4) from horse gram (*Macrotyloma uniflorum*) in peanut produced transgenic lines with better root system, improved biochemical and phsiological parameters, and increased antioxidant capacity contributing to enhanced drought tolerance (Pandurangaiah et al., 2014). Higher yield under water deficit conditions was also observed in transgenic peanut lines expressing the NAC2 gene from *A*. *thaliana* (Bhalani et al., 2019). Transgenic peanuts developed by expressing homeodomain-leucine zipper transcription factor (HDG11) from *A*. *thaliana* showed greater yield under drought and salt stress through upregulation of proline, antioxidant enzymes, stress-responsive genes, and enhanced physio-morphological traits (Banavath et al., 2018). Similar findings were noted when WRKY transcription factor from horse gram, MuWRKY3, was overexpressed in peanut (Kiranmai et al., 2018). Introduction of GmMYB3a, a MYB repressor protein from soybean (*Glycine max* L.) produced transgenic peanuts with efficient photosynthesis, higher WUE and RWC, indicating better adaptation in a water deficit condition (He et al., 2020). Overexpressing peanut bHLH transcription factor (AhHLH112) resulted in transgenic peanut lines with increase in antioxidant enzyme activity, ABA, and ABA-related genes and simultaneous reduction in ROS accumulation, MDA content, and water loss under deficit water conditions (Li et al., 2021).

Patil et al. (2014) overexpressed stress-responsive DNA helicase 45 (PDH45), a transcription activator from pea (*Pisum sativum*), controlled by 35 S promoter from the Cauliflower Mosaic Virus (CaMV35S) in a peanut genotype with superior water relation traits to pyramid drought tolerance traits. This led to the development of transgenic lines with improved water use efficiency, chlorophyll stability, better growth rate, rooting, and greater productivity compared to the wild type. Expression of eukaryotic translational initiation Factor 4A (eIF4A) gene from pearl millet (*Pennisetum glaucum)* using rd29A promoter in peanut produced transgenic lines with a better shoot and root growth, augmented ROS scavenging ability, and increased membrane stability in both drought and salt stress (Tiwari et al., 2015; Rao et al., 2017). Overexpression of the abscisic acid stress ripening-1 (ASR-1) gene from a halophyte, *Salicornia brachiate*, produced transgenic peanut lines with increased concentrations of beneficial metabolites such as sugar, proline, and starch under water stress. These lines also showed a reduction in the accumulation of malondialdehyde (MDA) and ROS, increasing drought tolerance (Tiwari et al., 2015). Overexpression of the KCS1 gene, a critical gene for epicuticular wax biosynthesis, from drought tolerant peanut cultivar into drought sensitive peanut cultivar under the control of CaMV35S promoter increased the deposition of wax on the leaves of transgenic lines preventing water loss (Lokesh et al., 2019). The transgenic lines also displayed increased proline, alcohols, fatty acids, aldehydes, alkanes, and ketones content and reduced oxidative damage and MDA content improving their drought tolerance.

Multiple transgenes have been stacked together to improve drought tolerance in peanuts. Pruthvi et al. (2014) simultaneously expressed three transcription factors, AtDREB2A, AtHB7, and AtABF3 that regulate downstream stress-related genes, from *A. thaliana* under CaMV35S promoter. These transgenic plants accumulated greater biomass than the wild type along with the production of higher amount of proline, expression of cellular tolerance genes, and their superior ability to scavenge ROS arising from stress. Similarly, co-expression of three regulatory genes related to drought tolerance traits: alfalfa zinc finger 1 (Alfin1), a transcription factor related to root growth under 35 S promoter, *P. glaucum* heat-shock factor (PgHSF4) under stress-inducible rd29A, and pea DNA helicase (PDH45) under 2 × 35SCaMV promoter produced transgenic peanut lines with better growth, elevated RWC, and enhanced expression of stress responsive genes indicating greater drought tolerance in transgenic lines than the wild-type peanut lines (Ramu et al., 2016). In another multigene transgenic study, three stress responsive transcription factors from horse gram were concurrently expressed: MuWRKY3, related to defense against ROS under CaMV2x35S promoter, MuNAC4, related to drought tolerance under ubiquitin promoter, and MuMYB96, related to cuticular wax synthesis under ribulose-1,5-bisphosphate carboxylase small subunit (rbcS) promoter (Venkatesh et al., 2022). These multigene transgenic plants displayed increased tolerance to drought than wild types through increased wax layer accumulation, better ROS scavenging, and greater shoot and root growth.

Apart from the overexpression of regulatory genes, transgenic approaches have been utilized to enhance drought tolerance through increase in solute concentration in the plant vacuoles. *Arabidopsis* vacuolar H + -pyrophosphatase gene (AVP1) associated with pumping protons on the vacuolar membrane was overexpressed in peanut under 35 S promoter (Qin et al., 2013). The resulting transgenic peanuts outperformed their wild type counterparts in photosynthetic rate, biomass production, and yield under water deficit conditions arising from drought or salt stress. Overexpressing vacuolar Na+/H+ antiporter gene (AtNHX1) under the control of 35 S promoter in peanut not only improved salt tolerance through build-up of salts and proline in leaf tissue but also the plants performed better upon drought stress (Asif et al., 2011). Overexpression of AhRabG3f gene, associated with lysosomes, under CaMV35s promoter in peanut enhanced their tolerance to drought stress through the expression of multiple stress-responsive genes (Song et al., 2012).

In addition to the plant genes, genes from bacteria have also been introduced in peanuts to enhance their response to drought stress. Qin et al. (2011) introduced isopentenyltransferase (IPT) gene, related to cytokinin synthesis, from *Agrobacterium tumefaciens* under the control of water-stress inducible senescence-associated receptor protein kinase (SARK) promoter from bean (*Phaseolus vulgaris*). They reported that transgenic peanut lines were significantly more drought tolerant than the wild types under drought stress in a greenhouse, growth chamber, and in the field conditions as manifested by their larger roots, higher transpiration rate, photosynthetic rate, stomatal conductance, and greater yield. The ability of *Escherichia coli* to convert fructose to mannitol, an important compound to mitigate abiotic stress, has been utilized by introducing mannitol-1-phosphate dehydrogenase (MtlD) gene into peanut (Bhauso et al., 2014). Transgenic peanuts expressing mtlD gene had better accumulation of compounds like mannitol and proline, higher chlorophyll content, RWC, and osmotic potential and reduced cell membrane leakage under salt and water stress condition. Patel et al. (2017) characterized the transgenic peanut lines developed by Bhauso et al. (2014) under water deficit conditions. They reported that the transgenic lines accumulated higher amounts of mannitol and suffered lower oxidative injuries, showed better photosynthetic performance, and yielded more than wild types. In summary, overexpression of multiple genes in peanuts has developed transgenic peanut lines with improved drought tolerance. However, no transgenic peanut lines have been released (Mallikarjuna et al., 2016). Bio-safety assessment, public perception and acceptance, and governmental policies will determine the release and successful adoption of transgenic and genome edited drought tolerant peanut lines (Singh et al., 2024).

## 5 Genetic resources and breeding tools

The cultivated peanut is an allotetraploid derived from the natural hybridization of its diploid progenitors, *A. duranensis* (A-subgenome) and *A. ipaensis* (B-subgenome). *A. hypogaea* has a strong linkage disequilibrium (LD) and limited genetic diversity (Bertioli et al., 2020). Following their domestication, cultivated peanuts spread worldwide, where intensive selection over generations led to narrow genetic variation. For instance, the Florunner cultivar, which can be traced to four founder lines, dominated the US market for two decades after its release in 1969 and was extensively used in many breeding programs (Norden et al., 1969). After the appearance of tomato spotted wilt virus (TSWV) in the southeastern US, a plant introduction (PI) line introduced from Brazil, PI 203396, contributed at least 25% of its genetics to most modern peanut cultivars (Clevenger et al., 2017). One way of mitigating this narrow genetic diversity in peanuts is by expanding variation through introgression from wild relatives possessing beneficial alleles.

The availability of germplasm collections, genomic resources, bioinformatics tools, and advanced technologies such as high throughput phenotyping facilitate characterization of diversity and breeding for desirable peanuts. Genome sequences of the cultivated peanuts, the A-genome and B-genome progenitors, and some other wild relatives have significantly contributed to peanut breeding programs (Bertioli et al., 2016; Bertioli et al., 2019; Bertioli et al., 2021). These resources are crucial for peanut breeders worldwide to evaluate breeding populations and to identify beneficial germplasm (Leal-Bertioli et al., 2009; Stalker, 2017; Dutra et al., 2018).

### 5.1 Utilization of wild relatives to improve drought tolerance

Wild relatives offer a diverse genetic pool that can be explored to improve drought tolerance in cultivated peanuts. Several genetic and transcriptomic studies have reported that the A-genome donor of cultivated peanuts (*A. duranensis*) exhibits tolerance to drought stress (Guimarães et al., 2012; Brasileiro et al., 2015; Thoppurathu et al., 2022). Leal-Bertioli et al. (2012) investigated key traits related to drought adaptation in the cultivated peanut progenitors, *A. duranensis* and *A. ipaensis,* and their derived synthetic allotetraploid. Both morphological (e.g., leaf thickness, trichome density) and physiological (e.g., transpiration profiles under water deficit conditions) traits at both ploidies were evaluated suggesting that morphological and physiological changes may occur upon hybridization and polyploidization. Cason et al. (2020) identified *A. dardani* as a potential donor for drought tolerance traits and analyzed differentially expressed transcription factors that may enhance the plant’s ability to withstand water deficit conditions.

Another wild peanut species, *A. stenosperm*a, was reported to be a potential donor for drought tolerance, as it possesses a candidate endochitinase-encoding gene (*AsECHI*), that enhanced post-drought recovery in transgenic *Arabidopsis* (Mota et al., 2021). However, a comparative transcriptome study between *A. stenosperma* and *A. duranensis* revealed that *A. duranensis* had a better drought tolerance than *A. stenosperma* (Vinson et al., 2018). The expression profiling of two wild peanut species *A. duranensis* and *A. magna* focused on transpiration rates and gene expression levels in roots and leaves under progressive water deficit conditions and identified genes such as Expansin, Nitrilase, NAC, and bZIP transcription factors differentially expressed during the drought stress (Brasileiro et al., 2015). While offering promising alleles for drought tolerance, wild peanuts are mostly diploid and often associated with linkage drag, complicating their introduction into breeding programs.

### 5.2 Development of drought tolerant peanut germplasm/cultivars

Germplasm screening for drought tolerance-related traits has been conducted by several researchers to identify accessions that can be used as sources of drought tolerance (Rucker et al., 1995; Upadhyaya, 2005; Bennett et al., 2022). In a series of greenhouse and field trials conducted by Rucker et al. (1995), two genotypes, Tifton 8 and PI 315628 were identified as high-yielding genotypes under water-limited conditions, exhibiting drought avoidance traits such as low visual stress rating, low canopy temperature, and large root systems. Highly drought-tolerant genotypes were also identified based on their drought susceptibility index (DSI), which included ICGV 91114, K 1375, and ICGV 02125 (Pavithradevi et al., 2015). Recently, a multi-parent advanced generation inter-cross (MAGIC) population was created for studying and developing drought tolerance peanuts. Derived from 8 parents, including ICGV 02022, ICG 7190, ICGV 97183, ICG 3053, ICG 14482, ICG 11515, TAG 24 and ICGV 0266, this population generated some drought tolerance advance lines that can serve as valuable resource for peanut breeding programs (Sharma et al., 2024). Sinclair et al. (2018) studied Virginia-type peanut germplasm to identify the genotype that can reduce its transpiration rate earlier when limited water is available in the soil, consequently conserving moisture at an early stage of drought. They found that genotype N12006ol had a high threshold for fraction of transpirable soil water (total available soil water/total transpirable soil water), delayed wilting, and higher yield than the control genotype “Bailey” under a limited water environment due to an early decrease in transpiration rate.

A genotype GP-NC WS 17 was found to be drought-tolerant, which maintained healthy leaves with low epidermal conductance and high SPAD chlorophyll content, SLA, and unsaturated/saturated fatty acid content under severe water deficit. Another genotype C431-1-1 demonstrated drought tolerance with high thermotolerance and maintained overall pigment concentrations under early drought stress (Tallury et al., 2014; Fabreti et al., 2018). Cheek (2024) also confirmed that GP-NC WS 17 and C431-1-1 possess both drought tolerance and reduced aflatoxin contamination. Cultivated peanut germplasms tolerant to drought stress from different countries, including the United States of America, China, and India, are summarized in Puppala et al. (2023). With the advancement in genomic studies, several genomic regions associated with drought tolerance have been identified and molecular markers have been developed to facilitate the selection process.

### 5.3 Identification of QTLs (quantitative trait loci) and marker development

Drought tolerance in peanuts is a quantitative trait resulting from the cumulative effects of multiple genes and interaction with the environment (Ravi et al., 2011). Advances of genotyping and sequencing technologies have enabled the discovery of QTLs and markers associated with drought tolerance in peanuts leading to marker-assisted selection (MAS). Such markers enable breeders to select drought-tolerance traits at early stages, accelerating the breeding cycle, and increasing the likelihood of developing successful drought-resistant cultivars. This approach has been adopted and has benefited several peanut breeding programs worldwide. A simple sequence repeat (SSR)-based linkage map for cultivated peanuts was first developed in 2009 using a RIL mapping population derived from ICGV 86031 × TAG 24 (Varshney et al., 2009). It was utilized to discover 2-5 QTLs each for surrogate traits for drought tolerance including transpiration, transpiration efficiency, SLA and SCMR, explaining 3.5%–14.1% of phenotypic variation. Axiom_Arachis SNP array version 1 (58 K) and version 2 (48 K) were developed in 2017 and 2018 using different cultivated peanut lines, their progenitors, and wild diploid species (Clevenger et al., 2017; Pandey et al., 2017; Clevenger et al., 2018; Korani et al., 2019). Genome-wide QTL analysis based on the Axiom_Arachis 58 K array identified 16 major main effect QTLs pertaining to drought tolerance (Pandey et al., 2021). Several candidate genes encoding transcription factors like MYB, NAM, MADS-box, bHLH, and other signaling and stress response-related genes were identified in these QTL regions. Fonceka et al. (2012) investigated a synthetic allotetraploid peanut population under well-watered and water-limiting conditions and discovered 13 significant QTLs for stress tolerance indices like total biomass, seed, haulm, and pod weight. Their study revealed that several QTLs were contributed by the amphidiploid parent highlighting the importance of using wild relatives for improving drought tolerance in breeding of cultivated peanuts.

In a genome-wide association study (GWAS) of 300 peanut genotypes, 68 marker-trait associations (MTAs) linked to traits such as leaf area, leaf dry weight, SPAD chlorophyll meter readings (SCMR), harvest index, haulm weight, and seed weight were found under limited water conditions, with phenotypic variation explained (PVE) ranging from 8.24% to 90.09% (Pandey et al., 2014). Shaibu et al. (2020) identified markers associated with traits including LAI, canopy temperature, SCMR, and NDVI, with marker related to SCMR explained the highest phenotypic variation of 20.8% while markers for other traits explained 6%–10% phenotypic variation. Abady et al. (2024) conducted a GWAS study on peanut populations in water-stressed and well-watered conditions. This led to the identification of 48 markers of which 47 were detected under drought and under non-stressed conditions. A single nucleotide polymorphism (SNP) was identified for each trait including leaf area, LAI, SLA, number of primary branches and 43 SNPs for relative water content in leaves. These SNPs were located within or near candidate genes includes *Araip.9NG64* (RNA-binding protein), *Aradu. PKW10* (CD2 antigen cytoplasmic tail-binding-like protein), *Araip57P4D* (Chitinase), *Araip.4J8RL* (Polynucleotide phosphatase/kinase), *Araip. SVH5H* (ABC transporter protein), *Aradu. VIU0I* (Zinc finger MYM-type 1-like protein), and *Aradu. ML3P3* (P-type ATPase), all of which are implicated in abiotic stress response and drought tolerance (Abady et al., 2024). Additionally, a GWAS conducted on a peanut MAGIC population developed for drought tolerance discovered 37 MTAs, most of which are located on chromosomes 03, 07, 10 and 18 (Sharma et al., 2024). Candidate genes in these regions were found to be associated with leaf senescence, flowering, chlorophyll biosynthesis, stomatal regulation, and yield-related traits.

Ravi et al. (2011) identified 105 and 65 main effect QTLs related to drought tolerance in peanuts by QTL Cartographer and QTLNetwork with PVE ranging between 1.3% and 33.36%, indicating that drought tolerance in peanuts is influenced by multiple small effect QTLs. This study evaluated a wide range of traits related to drought tolerance, including transpiration, transpiration efficiency, SLA, leaf area, SCMR, carbon isotope discrimination ratio, biomass, canopy conductance, total dry matter, dry weight, pod weight, seed weight, and haulm weight. Gautami et al. (2012) used three RIL populations and identified 153 main effect QTL and 25 epistatic QTLs. These QTLs were associated with important drought-related traits, such as transpiration efficiency, transpiration, total dry weight, shoot dry weight, SCMR, HI, and vegetative weight per plant (VegWt/pL) but none of them were major QTLs (Varshney et al., 2009; Ravi et al., 2011). Faye et al. (2015) identified 52 QTLs with low effects, each explaining less than 12% of the phenotypic variance for nine yield-related traits and SPAD chlorophyll meter readings. As multiple studies found small effect QTLs for drought tolerance in peanuts, genomic selection has been proposed as an alternative strategy to marker-assisted backcrossing and marker-assisted recurrent selection (MARS) to breed drought-tolerant peanut varieties (Ravi et al., 2011; Faye et al., 2015).

## 6 High throughput phenotyping

High-throughput phenotyping (HTP) technologies have become more accessible and started being adopted in peanut breeding over the past few years. Although still under developmental phase, HTP platforms hold potential for accelerating the breeding process by enabling rapid and precise trait assessment (Balota and Oakes, 2017; Sarkar et al., 2021; Balota et al., 2024). Conventional phenotyping methods in peanut breeding involve manual measurement using specialized devices to evaluate plant responses to drought stress. Manual assessment methods commonly used in peanuts include visual ratings, specific leaf area, specific dry weight, total dry matter content, pod yield, chlorophyll fluorescence, SPAD chlorophyll meter reading, normalized difference vegetation index, canopy temperature, relative water content, photosynthesis rate, stomatal conductance, and so on (Upadhyaya, 2005; Sullivan and Holbrook, 2007; Luis et al., 2016; Buezo et al., 2019; Ghosh et al., 2022; Zhang et al., 2022a). These methods are laborious, time inefficient, allow only a limited number of genotypes assessment, and are prone to inconsistency (Baslam et al., 2020; Kim, 2020; Jangra et al., 2021). Automated data collection can reduce labor costs and increase the throughput of phenotypic evaluations, enabling breeders to screen larger populations more efficiently. Puppala et al. (2023) summarized recent HTP technologies that have been adopted for phenotyping drought-associated traits in peanuts, including the utilization of sensors such as RGB (red, green, blue), multispectral, hyperspectral, thermal, LiDAR (light detection and ranging) and GPR (ground penetrating radar). Unmanned aerial vehicles (UAV) with RGB, multispectral, and hyperspectral sensors can estimate various drought-tolerance-related traits in peanuts including leaf wilting rating, leaf area index, and biomass once correlations with ground-truth data are established (Balota and Oakes, 2017; Sarkar et al., 2020; Sarkar et al., 2021). Other platforms including pushcart, minirhizotron, handheld, and UGV (unmanned ground vehicle) with various sensors that can measure pod yield, root architecture, transpiration, and plant height have also been implemented in peanut (Balota and Oakes, 2017; Yuan et al., 2019; Xu et al., 2020; Bidese Puhl et al., 2021; Dobreva et al., 2021). Combining UAV-based hyperspectral imaging and machine learning, a model was created that can predict different traits efficiently under drought stress (Bagherian et al., 2023). Balota et al. (2024) investigated 21 peanut accessions from the US peanut mini-core germplasm collection to identify performance in rainfed and limited water conditions using drone and manual measurements. The correlation between manual and aerial evaluation ranged from 0.02 to 0.94, where traits such as CO_2_ assimilation, stomatal conductance and transpiration rates had a higher correlation between manual and aerial analysis. High throughput phenotyping holds immense potential for drought evaluation. As technology advances, HTP can be more reliable than conventional phenotyping methods, offering more efficient and precise measurement.

## 7 Future direction

Drought is a polygenic and complex trait. The complexity of studying drought stress is further exacerbated in field-based research due to the differences in soil type, soil moisture, weather conditions, and rainfall. As a result, a variety released as a drought-tolerant variety in one location may not perform well in another due to strong genotype by environment interaction. This strong environmental impact also complicates the genetic dissection of drought tolerance.

Phenotyping drought stress in peanuts presents significant challenges. Various methods have been implemented to evaluate drought-related stress responses, including the measurements of leaf water potential, stomatal conductance, relative water content, chlorophyll content, hydraulic conductance, and investigating root growth parameters. While these traits are vital to understanding the physiological response to drought, they often require significant labor and time to measure.

Tools to measure such physiological traits are anticipated to become more user-friendly, precise, and less labor demanding. The advent of UAV imaging and field sensors has significantly improved the efficiency drought tolerance studies (Bagherian, 2022; Balota et al., 2024). The use of aerial sensing will allow plant breeders to perform more efficient artificial selection from large populations. Soil moisture content and temperature play vital roles in evaluating drought stress. Sensor-based irrigation systems, remote sensing, and geographic information systems (GIS) will be common implements for drought studies in the future.

One of the major challenges in drought studies is to evaluate below-ground root growth. Imaging tools such as minirhizotron system (Vienna Scientific Instruments), ground penetrating radar (GPR) (Liu X. et al., 2018), and magnetic resonance imaging (MRI) (van Dusschoten et al., 2016), combined with artificial intelligence (AI) may offer promising solutions for conducting peanut root studies under drought conditions. Another complexity in peanut root-system study is that the economic yield component (pod) also lies below ground. Peanuts bloom above ground but when flowers are fertilized, a peg is formed, which starts growing underground and forms fruit below the soil surface. In studying peanut yield or root growth using non-destructive methods, it is vital to distinguish between roots and pods and some advancement has already been made (Gimode et al., 2023).

With sequencing technologies becoming more precise and the costs decreasing exponentially, marker-assisted selection is expected to become widely utilized in peanut breeding programs. It will be more common to observe the amalgamation of genetics and data science as sequencing becomes routine work for drought studies. These advancements will facilitate predictive breeding and genomic selection to develop drought-tolerant peanut cultivars. Simulated drought studies, machine learning, AI, and modeling of environmental, genomic and phenomics data will be commonly integrated with drought studies.

The availability of extensive phenotypic data and error-free sequencing technology enables precise discovery of genomic regions conferring drought tolerance in peanuts. Such discoveries could be facilitated by the construction of several pangenomes as reference genomes in peanuts. A pangenome can represent a complete set of genes present in a species and can capture structural variants and genetic diversity better than a single reference approach (Danilevicz et al., 2020).

Although not for drought studies, CRISPR/Cas9 gene editing has been successfully deployed in peanuts (Li et al., 2023; Conner et al., 2024). As we precisely identify genetic loci, CRISPR technology could potentially edit genes associated with drought stress. Advancements in the use of such multi-omics and gene editing technologies will expand the opportunities to exploit variation to improve drought tolerance.

Genetic variation is a key to breeding successful cultivars in any crop. Cultivated peanuts have a very narrow genetic base and a strong linkage disequilibrium which challenges breeding for complex traits like drought tolerance. Exploration and conservation of wild and local landraces of peanuts is critical. Several approaches to broaden the peanut genetic base that include MAGIC, nested association mapping (NAM), mutant line development, and wild introgression will continue to have their space in future peanut genetic improvement.
